# Coronary artery bypass grafting for an octogenarian with R II‐B type with isolated single coronary artery

**DOI:** 10.1002/ccr3.2602

**Published:** 2019-12-09

**Authors:** Motoyuki Kumagai, Eiji Shinoda, Makoto Takehara, Junichiro Nishizawa

**Affiliations:** ^1^ Department of Cardiovascular Surgery Hamamatsu Rosai Hospital Hamamatsu Japan; ^2^ Department of Cardiology Hamamatsu Rosai Hospital Hamamatsu Japan

**Keywords:** CABG, coronary artery anomalies, isolated single coronary, octogenarian

## Abstract

We experienced a very rare case of isolated single coronary artery, in which the left main coronary artery passes between the aorta and pulmonary artery. It is the most potentially serious among the coronary artery anomalies, because it has the risk of myocardial infarction and sudden death in young ages.

## Graphical abstract

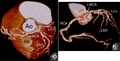

## 1 INTRODUCTION

**Figure 1:**
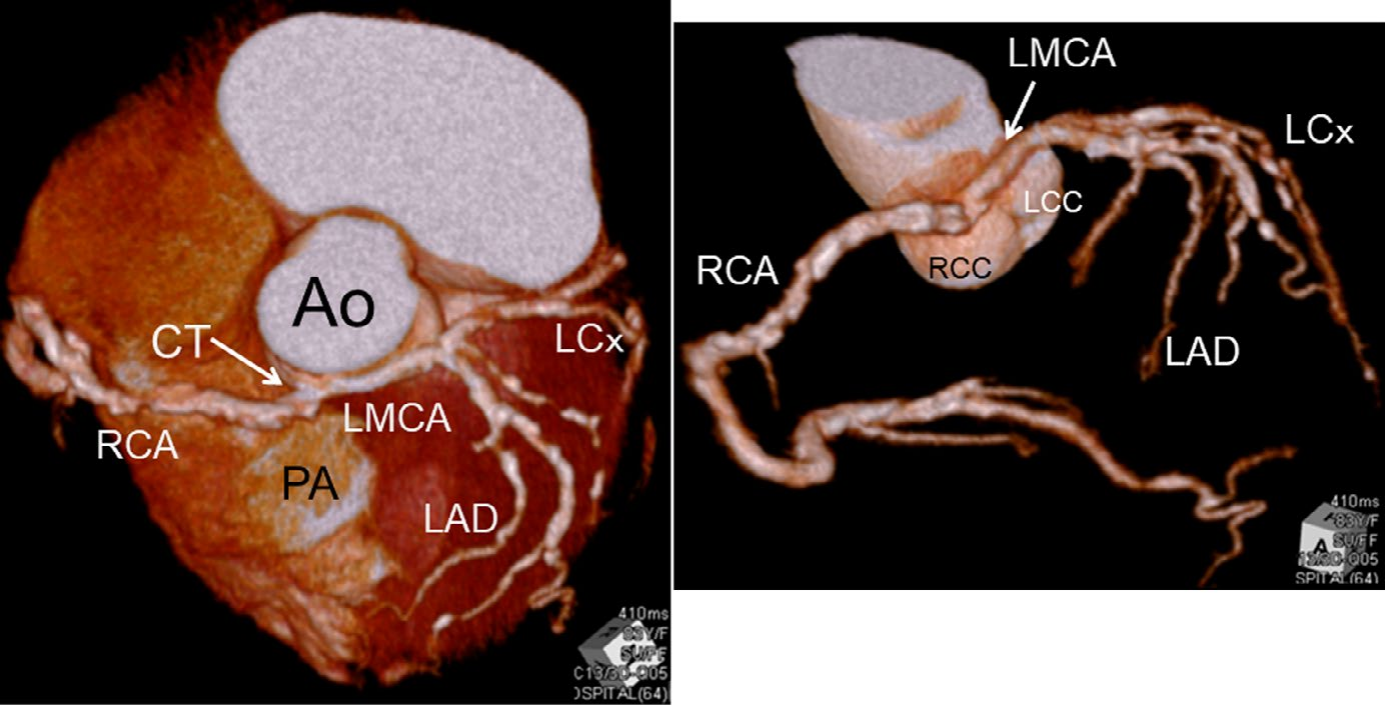
Cardiac multidetector‐row computed tomography findings. Coronary arteries arise from single ostium at right sinus of Valsalva, and left main coronary artery runs between aorta and pulmonary artery. Ao, Ascending aorta; CT, short common trunk; LAD, left anterior descending coronary artery; LCC, left coronary cusp; LCx, Left circumflex artery; LMCA, left main coronary artery; PA, pulmonary artery; RCA, right coronary artery; RCC, right coronary cusp

An 83‐year‐old woman was admitted with angina pectoris. Multidetector‐row computed tomography (MDCT) and coronary arteriography revealed an isolated single coronary artery with other coronary arteries arising from a single ostium at the right sinus of Valsalva. The left main coronary artery ran between the aorta and pulmonary artery (R II‐B type). Calcified two vessel coronary artery disease and angina pectoris were evident, with 90% stenosis in the left anterior descending artery (LAD), the diagonal branch, and the left circumflex artery (LCx) (Figure 1). Coronary artery bypass graft surgery proceeded with a cardiopulmonary bypass under cardiac arrest as follows. The LAD was grafted to the left internal thoracic artery, and the LCX and the diagonal branch were bypassed with a saphenous vein graft. The patient was discharged on postoperative day 55 without major adverse events.

An R II‐B type isolated single coronary artery is very rare, with an estimated incidence of 0.015%. However, it was the most potentially serious among the coronary artery anomalies, because it is associated with high risk of sudden death in the absence of coronary atherosclerosis in younger patients.^1^ Our patient is very unusual because she remained asymptomatic until she reached the age of 83 years, and not all patients who had the R II‐B type isolated single coronary may be very dangerous.

## CONFLICT OF INTEREST

None declared.

## AUTHOR CONTRIBUTIONS

MK: treated the patient, captured the images, coconducted the literature review, and cowrote the paper. ES: analyzed the case, cowrote the paper. MK: treated the patient. NJ: treated the patient and corrected the paper.
